# Essentials of Histology

**Published:** 1907-05-11

**Authors:** E. A. Schäfer

**Affiliations:** University, Edinburgh


### Editor's Letter-Box.

### "ESSENTIALS OF HISTOLOGY."

Sir,—It is not my custom to openly notice criticisms of my books, although I am always glad to adopt improvements which my critics may suggest. But the review, in your number of February 23—which has only just come into my hands—of the seventh edition of my "Essentials of Histology," although as a whole appreciative, contains certain errors of fact and suggestion which it seems not undesirable to indicate.

Your reviewer especially complains that the directions for much of the practical work are "given with so little detail that it would be very difficult for a student, working by the book alone without an instructor, to follow them." He should, however, have been aware, as he speaks with apparent familiarity of former editions, that this book was never intended for independent practical work, but solely for class work, under the guidance of an experienced teacher. He must know that it would be impossible to introduce within the limits of a small handbook the innumerable details upon the precise attention to which success in the carrying out of histological methods can alone be attained. This has been definitely stated in the preface to every preceding edition, and it was only omitted from this edition because I was of opinion that a fact so obvious did not need further accentuation. I now perceive I was mistaken. Nevertheless the references in the preface of this edition to the "class," the "teacher," and the "lesson" might, I think, have suggested to my critic that he was adopting an erroneous view of the object of the directions, which are given. And the criticism which he bases upon this view would then have seemed to him, as it does to me, to be superfluous.

He further complains that some of the illustrations are "quite poor," but is, I think, unfortunate in the three instances which he has selected in support of this statement. The quality of an illustration is naturally a matter of opinion, but I feel sure most histologists will agree with me that no more exact representation of detached epithelium scales from the mouth could well be furnished than that given in fig. 54. This is, it is true, an old figure: I believe from the pencil of Sharpey; but I know none better. Why it should be "pardoned for being a teased preparation," and what it has to do with "lymph glands," in connection with which it is mentioned by your reviewer, I am unable to understand.

The exception taken to the accurate and beautiful figure, from V. Ebner, of a low power view of a section through a human lymph gland (fig. 253), is still less comprehensible. "No student could be expected to learn to recognise lymphatic gland from it. Were it not for the description in the text, it might be taken for a developing bone." As it is evident that your reviewer is not familiar with the appearance of a section of human lymph gland, I am not prepared to contest his statement that he might take it for a developing bone (!); but I do not hesitate to say that few students would fail to recognise its true nature or its close correspondence with the appearance of an actual specimen. The third instance given is the figure of a section of elastic ligament. "Fig. 86, again, professes to represent elastic fibres in cross section, but it might be a piece of adipo-fibrous tissue: it gives no idea of the sharp angularity that elastic fibres in cross-section present." I do not know what adipo-fibrous tissue is, and I cannot therefore say if this figure might represent it. That elastic-fibres, especially when closely packed, are often sharply angular is a familiar fact. But it is not an essential character, and their angles are often absent or much rounded as represented in the figure to which exception is taken. The illustration was in fact drawn from a carefully selected specimen by Mr. Richard Muir, perhaps the most able microscopic draughtsman we have; and, even without my own assurance, this fact may be taken as a guarantee that the drawing accurately represents the appearance of the specimen.

As to the merits of the Oliver method of rapidly estimating the amount of coloured corpuscles in blood, of Gowers' as compared witr Thoma's hœmacytometer, of photographs of sections of the central nervous system as compared with diagrams, there may be a difference of opinion, and mine is perhaps of no greater value than that of your reviewer. There can, however, be no such difference of opinion regarding the fact that the addition of sodium iodide to blood is not necessary for the production of hæmin crystals. I am aware that this addition has been recommended by Carlier on the ground, *inter alia*, that larger crystals are thereby produced. But since the crystals are very easily obtained without it, it does not seem desirable to introduce the complication into an elementary text book, for which as far as possible the simplest efficient methods should be chosen. I therefore consider that this criticism also is not justified.

Yours faithfully,

University, Edinburgh, April 25. E. A. Schäfer.

